# Adherence to the Antibiotic Prophylaxis Guidelines for Appendectomy in Bahrain: An Observational Study

**DOI:** 10.7759/cureus.36975

**Published:** 2023-03-31

**Authors:** Ahmed K Alanzi, Shahid Adeel, Samah Hakmi, Amer AlDerazi

**Affiliations:** 1 Anesthesia and Critical Care, King Hamad University Hospital, Muharraq, BHR; 2 Surgery - General and Bariatric Surgery, Salmaniya Medical Complex, Manama, BHR

**Keywords:** anesthesia, prophylaxis, appendectomy, surgery, antibiotics, appendicitis

## Abstract

Background

Appendicitis is a common clinical problem that has multifactorial etiologies. Accounting for almost 1 million hospital days per year, it poses serious health hazards. If not treated on time, it may burst. Surgical intervention is the best option in such cases. Prophylactic use of antibiotics has been shown to reduce post-operative infections.

Methodology

This prospective observational study aimed to evaluate the adherence to the antibiotic prophylaxis guidelines for appendectomy in patients admitted to the surgical department at Salmanyia Medical Complex in Bahrain from January to August 2020. From the electronic records of these patients, information was extracted and evaluated regarding demographic data, the type of antibiotics given for prophylaxis, the timing of the administration of the antibiotics, and any alternative antibiotic given based on local hospital guidelines.

Results

The current study revealed that the majority of the patients (98%, N=273) admitted to the Salmanyia Medical Complex, Bahrain, were not administered the antibiotics within the prescribed time (30-60 minutes) as per hospital guidelines. Also, the antibiotics administered for prophylaxis prior to the appendectomy procedure were not according to the guidelines, i.e., Cefazolin 1g with Metronidazole 500 mg. Out of a total of 278 patients included in the study, none were administered the right choice as provided by the local guidelines. Second, 1.8% of patients (5 out of 278) were not administered any antibiotics for prophylaxis prior to the surgical procedure for appendicitis.

Conclusion

The study concluded that most patients were not administered antibiotics according to the local guidelines of the hospital.

## Introduction

The incidence of appendicitis is common among both young and pediatric populations. The lifetime risk for males is 8.6% and 6.7% for females [1]. Appendicitis remains the most prevalent cause of abdominal discomfort across the globe. Appendectomy is not a common surgical emergency; acute abdominal surgeries are the most common general surgical emergency, and acute appendicitis causes acute abdomen [2]. Although the appendix's precise role has been a source of contention, recent research acknowledges its immunoprotective role, particularly in the young. Alternatively, other ideas assert that the appendix serves as a reservoir for "healthy" intestinal bacteria. Others think that it is only a developmental residue with no discernible function [2-4]. Typical clinical symptoms are observed in 50-60% of patients with appendicitis [5,6]. The clinical diagnosis of acute appendicitis is accurate in up to 80% of cases.

Although appendicitis is a commonly reported healthcare problem, the diagnosis is challenging. The frequency of needless appendectomies due to a false-positive clinical diagnosis ranges between 13% and 30%, with a mean false-negative appendectomy rate of around 20% before imaging. False-negative appendectomy rates in female patients aged 10 to 39 years are as high as 15-47% [7,8]. Appendicitis symptoms are usually easy to recognize by a healthcare provider. Abdominal pain is the most prevalent sign of appendicitis. Appendicitis causes abdominal pain that starts near the belly button and spreads lower and to the right, often waking a person up in the middle of the night before additional symptoms appear. Other appendicitis symptoms include loss of appetite, nausea, vomiting, constipation or diarrhea, inability to pass gas, low-grade fever that occurs after other symptoms, stomach swelling, and the expectation that passing feces may alleviate pain.

The Alvarado score, Pediatric Appendicitis Score, and Appendicitis Inflammatory Response score incorporate common clinical and laboratory findings to stratify patients as low, moderate, or high risk and can help in making a timely diagnosis [9]. Recent advancements in diagnostic imaging, especially computed tomography (CT), have enabled a more precise diagnosis of appendicitis [10]. The increased use of CT scanning and laparoscopy has increased the number of instances of appendicitis diagnosed, resulting in a higher appendectomy rate, suggesting that the illness may recover spontaneously [11]. While antibiotics may be used as the primary final treatment in certain patients with suspected uncomplicated appendicitis, appendectomy remains the gold standard [12].

Before undergoing appendectomy, empirical therapy with broad-spectrum antibiotics is recommended to avoid infections within an hour before surgery [13]. A single dose of broad-spectrum antibiotics administered preoperatively (within 60 minutes prior to the surgical skin incision), as recommended by the World Society of Emergency Surgery (WSES), has been shown to be effective in reducing wound infection and post-operative intra-abdominal abscess, with no obvious difference in the nature of the separated appendix [14]. Preoperative administration of antibiotics in children with appendicitis is also recommended by the American Pediatric Surgical Association [15]. National Health Service (NHS), England also highly recommends the preoperative prophylaxis of antibiotics within one hour prior to the incision in appendectomy with intravenous Gentamicin and Metronidazole [16]. The current protocol at Salmaniya Medical Complex in Bahrain recommends that patients who present with acute appendicitis and require appendectomy receive antimicrobial prophylaxis 30-60 minutes prior to surgical incision. The current study was conducted to investigate adherence to antibiotic prophylaxis in appendectomy patients. This article was previously presented as eposter at the Association of Surgeons of Great Britain and Ireland in May 2022. The abstract was published as a poster at the 20th Annual QI and Audit Symposium at the Royal College of Surgeons of Edinburgh's 20th Annual QI and Audit Symposium on March 25, 2022.

## Materials and methods

Study design and target population

A prospective audit was undertaken at the Salmanya Medical Complex in Bahrain in 2020. The Ethics Committee of the Salmanya Medical Complex in Bahrain approved the audit protocol. Patients aged ≥18 years who underwent appendectomy (emergency and elective) were included in the study. Patients who received treatment doses of antibiotics against infection were excluded.

Study conduct

Data were obtained from the patients’ charts and entered into the data collection form. Information regarding demographic status, types of antibiotics recommended, alternate antibiotics administered, types of surgery used, nationality, and gender. According to the local guideline and policy for antimicrobial prophylaxis for appendectomy in Salmanya Medical Complex, Bahrain, all patients admitted to the hospital or presenting acutely to the hospital for appendectomy should be given antimicrobial prophylaxis within 30-60 minutes prior to surgical incision. The risks and benefits of antimicrobial antibiotics were discussed with the patients. As per guidelines, the antibiotics of choice for prophylaxis included cefazolin 1 g and metronidazole 1 g.

Compliance with the recommendations of the Salmanya Medical Complex was assessed in the study. The first criterion evaluated was whether antibiotics were given within the prescribed time following the hospital guidelines or not. The criteria were assessed as "appropriate" if antibiotics were given within 30-60 minutes before the procedure. The dose and route of administration of antibiotics were also recorded. The second criterion for evaluation was the choice of prophylactic antibiotics.

Statistical analysis

All statistical analyses were performed using SPSS version 20 (SPSS, Inc., Chicago, IL, USA). Frequency and percentage were calculated and presented.

## Results

**Figure 1:**
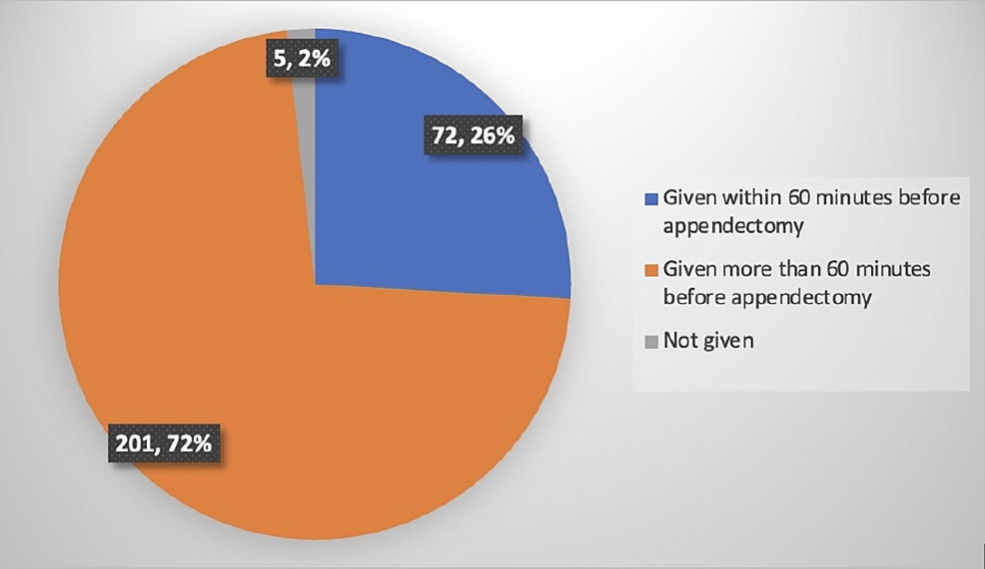
Timing of antibiotic administration

The records of a total of 278 patients were evaluated for gathering the required information. Patients involved in the study were from 18 to 57 years old (mean age 42.8 ± 15.2). A total of 273 (98.2%) patients were administered antibiotics as prophylaxis before surgery. Out of these 273 patients, 72 (26.37%) were administered antibiotics within or equal to 60 minutes prior to the surgical procedure, while the remaining 201 (73.63%) were administered antibiotics more than 60 minutes before the surgical procedure (Figure 1). The dose rate was appropriate in 33% of procedures (Table 1). All the antibiotics were administered intravenously.

**Table 1:** Compliance with the recommendations of the Salmanya medical complex therapeutic guidelines on antimicrobial prophylaxis in appendicitis (%)

Variables	Value
Appropriate agent used	12%
Appropriate initiation time of prophylaxis	26.37%
Appropriate dose	33%
Appropriate administration route	49%

**Figure 2:**
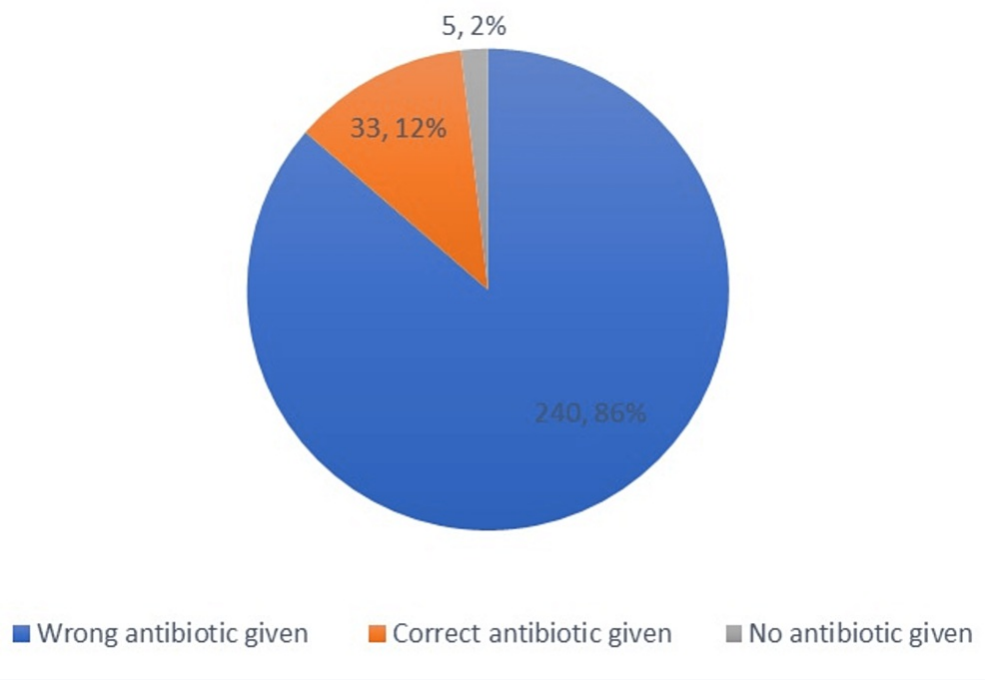
Choices of antibiotics for prophylaxis

Of the total of 278 patients, 273 (98.2%) were administered antibiotics, while 5 (1.8%) were not administered antibiotics for prophylaxis prior to surgery. Of the 273 patients who were administered antibiotics, 12% were administered the correct antibiotics as per the hospital's guidelines (Figure 2). The most frequently used antibiotics were cefazolin 56%, ceftizoxime 17%, clindamycin 10%, ceftriaxone 9%, and vancomycin 8%. Of 98.2% of participants, 21% received two or more antibiotics.

## Discussion

The current study revealed that the majority of the patients (98%, N=273) admitted to the Salmanya Medical Complex, Bahrain, were not administered the antibiotics within the prescribed time as per guidelines from the hospital and also that the antibiotics administered for prophylaxis prior to the appendectomy procedure were not according to the guidelines, i.e., cefazolin 1 g with metronidazole 500 mg. Out of the total of 278 patients included in the study, 12% were administered with the right choice as provided by the local guidelines. Second, 1.8% of patients were not administered any of the antibiotics for prophylaxis prior to the surgical procedure for appendicitis.

The preoperative administration of antibiotics has been recommended by various organizations, and positive outcomes of such prophylaxis have also been reported [13-16]. In 2001, a Cochrane meta-analysis concluded that preoperative broad-spectrum antibiotics are efficient in decreasing surgical site infections and abscesses. We analyzed randomized controlled trials and non-randomized comparative studies in which any antibiotic regimen was compared to a placebo in patients undergoing an appendectomy. This review included 44 studies involving more than nine thousand patients. Antibiotics were better than placebo in avoiding wound infection and intra-abdominal abscess, despite no obvious difference in the type of appendix excised [17].

Similarly, a systemic review by Daskalakis et al. also revealed the importance of the pre-operative administration of antibiotics in appendectomy. They reported that using broad-spectrum antibiotics prior to surgery seems to help with the rate of wound infection and abscesses. In this meta-analysis, 45 papers were utilized, and it was shown that, regardless of whether provided prophylactically or repetitively, antibiotics decrease the number of infection complications. The team of Almqvist et al. conducted a prospective randomized study on patients with gangrenous appendicitis, and their findings indicated that the timing of antibiotic therapy prior to surgery results in a statistically significant reduction in infectious complications as compared to that during the surgery [18].

The choice of antibiotics is important to cover a broad spectrum of bacteria in order to avoid infection. The effect of various antibiotic regimens on prophylaxis for appendicitis has been evaluated in four RCTs. Regarding the appendicitis population, a single dose of IV metronidazole before surgery was not as efficacious as a single dose of oral metronidazole [18]. Using cefotaxime and metronidazole, which were the most effective antibiotics, Kumarakrishnan et al. discovered that the combination had the lowest wound infection rate compared to metronidazole and ciprofloxacin [19], and according to the study of Liberman et al., single-dose cefotetan was as effective as multiple-dose cefoxitin [20].

A Cochrane meta-analysis also showed that the most commonly used antibiotics for prophylaxis prior to appendectomy were cephalosporins and derivatives of the imidazole group. Different studies have supported the combination for prophylaxis. A similar combination of imidazole and cephalosporin antibiotics (metronidazole 500 mg + cefazolin 1g) has been included in the local guidelines of the Salmanya Medical Complex, Bahrain, but was not being followed as observed in the current study. The study has some limitations, including a single-center study and a smaller sample size. Additionally, local guidelines might vary from hospital to hospital, so the result might not apply to many other centers. Although we investigated the appropriate use of antibiotics in appendectomy patients, the study did not measure the outcomes of delayed or skipped antibiotic therapy. Further research needs to be done to evaluate this aspect of antibiotic prophylaxis as well.

## Conclusions

The use of preoperative antibiotic prophylaxis in the case of an appendectomy has a significant effect on the reduction of abscesses and surgical site infections. The standard set by the Salmanya Medical Complex in Bahrain for antibiotic prophylaxis within 30 to 60 minutes with metronidazole 500 mg and cefazolin 1 g combination was not being followed for appendectomy. The current study recommends the proper implication of the standard guidelines regarding appendectomy procedures in order to achieve desired therapeutic outcomes with minimum complications.
